# Photothermal Synergistic Hydrogen Production via a Fly‐Ash‐made Interfacial Vaporific System

**DOI:** 10.1002/advs.202410201

**Published:** 2024-11-28

**Authors:** Yin Xie, Chenyu Xu, Yan Liu, Entao Zhang, Ziying Chen, Xiaopeng Zhan, Guangyu Deng, Yuan Gao, Yanwei Zhang

**Affiliations:** ^1^ State Key Laboratory of Clean Energy Utilization Zhejiang University Hangzhou 310027 China

**Keywords:** fly ash cenospheres, hydrogen production, interfacial evaporation, overall water splitting, photothermal catalysis

## Abstract

Employing UV–vis spectrum for hydrogen generation and vis‐IR spectrum to elevate reaction temperatures and induce phase transitions effectively enhances yield and purifies water, demonstrating a judicious strategy for solar energy utilization. This study presents an interfacial photothermal water splitting system that utilizes all‐inorganic, economical industrial by‐products known as fly ash cenospheres (FAC) for solar‐driven hydrogen generation. In this system, the yield reaches 254.8 µmol h^−1^ cm^−1^, representing an 89% augmentation compared to that of the three‐phase system. In situ experiments, combined with theoretical calculation, reveal the system's robust light absorption capacity, facilitating rapid gas separation, thus improves the solar‐to‐hydrogen (STH) efficiency. Furthermore, the system demonstrates strong performance in turbid water and scalability for expansive applications, achieving a hydrogen yield exceeding 50 L h^−1^ m^−2^ from various water sources. Facilitating large‐scale hydrogen production and water purification, it thereby establishing its potential as a viable solution for sustainable energy generation.

## Graphical abstract

This study proposes an interfacial photothermal system utilizing fly ash cenospheres (FAC) for solar‐driven hydrogen generation, achieving a yield of 254.8 µmol h^−1^ cm^−2^. The system demonstrates robust light absorption, rapid gas separation, and improved solar‐to‐hydrogen efficiency. It also shows scalability for water purification and large‐scale hydrogen production, making it a viable solution for sustainable energy generation.

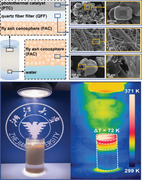

## 1 Introduction

Photothermal synergistic catalytic water splitting technology enables the direct harnessing of the full solar spectrum for hydrogen production within an integrated spatiotemporal framework, presenting an efficient and sustainable method for solar energy conversion.^[^ ^1^ ^]^ This strategy permits the coordinated use of both high‐quality (UV–vis) and low‐quality (vis‐IR) solar spectra, surmounting the constraints associated with single‐mode photothermal applications.^[^ ^2^ ^]^ Through the synergistic combination of photo‐ and thermo‐chemical contributions of sunlight, photothermal catalysis has the potential to enhance reaction rates and to change selectivity patterns, even under moderate operation conditions.^[^ ^3^ ^]^ By integrating with other heat‐intensive technologies, such as interfacial evaporation, this approach achieves extensive utilization of the solar spectrum, unveiling significant potential applications.^[^ ^4^ ^]^

Recently, solar‐driven interfacial evaporation, which transforms and concentrates solar heat at the air/liquid interface, has surfaced as an alternative to conventional bulk heating evaporation, providing decreased thermal losses and increased energy conversion efficiency.^[^ ^5^ ^]^ However, at present, there are few relevant studies on temperature‐resistant and thermal insulation materials that can be suspended on the water surface, mainly wood,^[^ ^6^ ^]^ aerogel/hydrogel,^[^ ^7^ ^]^ and other composite materials.^[^ ^8^ ^]^ These materials generally require a complex pretreatment process and have a relatively high cost. Due to their specific structure, there are challenges in experiments at high temperatures.^[^ ^9^ ^]^ Additionally, the mechanism of interfacial photothermal catalysis under high light intensity is not well understood, and further research is needed to determine how to promote the process of water decomposition. Coal combustion in power plants yields several by‐products, notably fly ash cenospheres (FAC), which are silicon‐alumina micro‐glass particles derived from fly ash.^[^ ^10^ ^]^ Composed predominantly of SiO_2_ and Al_2_O_3_, FAC possess a low density that allows them to remain buoyant in water. Their micrometer‐scale particle dimensions confer excellent water permeability.^[^ ^11^ ^]^ The low thermal conductivity of FAC aids in heat concentration at the surface, while their resistance to temperatures up to 1873 K renders them apt for high‐temperature reactions.^[^ ^12^ ^]^ The worldwide annual production of fly ash exceeds 900 million tons and shows an increasing trend, with fly ash cenospheres constituting 1% to 2% of the total.^[^ ^13^ ^]^ Such properties underscore the considerable potential of FAC for interfacial photothermal evaporation applications.

In this research, cost‐effective and eco‐friendly industrial byproducts, fly ash cenospheres (FAC), were utilized to create a photothermal evaporation interface, thereby enhancing photothermal water splitting. Rh, Cr, and Co‐loaded Al‐doped SrTiO_3_ as the prepared photothermal catalysts (PTC), which were uniformly dispersed on quartz fiber filters (QFF), and the PTC/QFF/FAC interfacial photothermal system was assembled. Utilizing solar energy exclusively, this system combines catalysis with heat accumulation at the interface to effectuate photothermal water splitting, achieving a hydrogen production rate of up to 254.8 µmol h^−1^ cm^−2^. Performance evaluations under high light intensities indicated enhanced catalytic efficiency. Characterization and thermodynamic simulations confirmed that concentrated solar irradiation improves catalytic activity and product transfer rates, hence boosting hydrogen production and water evaporation. Scaled experiments achieves a hydrogen yield exceeding 50 L h^−1^ m^−2^ from various water sources, demonstrating the system's capability to produce hydrogen and purify water, effectively supporting its potential for energy conversion, storage, and scalability in large‐scale applications. This approach, coupled with its carbon emission reduction potential, positions it as a formidable contender for sustainable energy production.

## 2 Results

### 2.1 Overview of the PTC/QFF/FAC System

**Figure 1:**
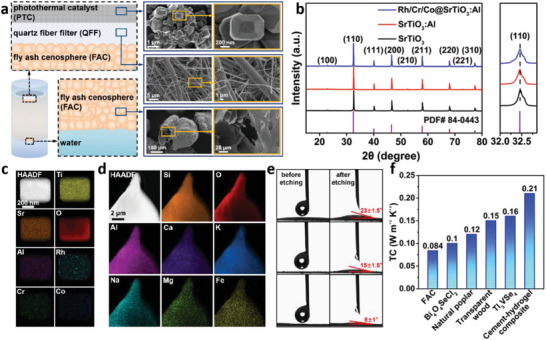
The designed PTC/QFF/FAC system and characterization of materials. a) Schematic diagram of the manufacturing process of an efficient interface photothermal reaction system, with SEM images of PTC (top), QFF (middle), and FAC (bottom) on the right. b) XRD image of the Rh, Cr, and Co‐loaded Al‐doped SrTiO_3_ photothermal catalysts (PTC). c) TEM images and EDS element mapping of PTC. d) TEM images and EDS element mapping of fly ash cenospheres (FAC). e) Water contact angle test images of original FAC (top), FAC after washing (middle), and FAC after calcination (bottom). f) Comparison of thermal conductivity between FAC and other recently reported thermal insulating materials (Bi_4_O_4_SeCl_2_,^[^ ^15^ ^]^ natural poplar,^[^ ^16^ ^]^ transparent wood,^[^ ^17^ ^]^ Tl_3_VSe_4_,^[^ ^18^ ^]^ and cement‐hydrogel composite^[^ ^19^ ^]^).

The PTC/QFF/FAC system is structured in a three‐tiered architecture, comprising distinct functional layers (**Figure** **1**a). Constructing this system requires preparing photothermal catalyst, quartz fiber filters, and fly ash cenospheres. The uppermost layer functions as the photocatalytic layer, whereas the QFF acts as the heat‐collecting and catalyst‐supporting intermediate layer. Below these, the FAC serves as both a water‐transporting and heat‐insulating layer. In the interfacial photothermal synergistic reaction, each functional layer fulfills its specific role concurrently. The PTC layer directly absorbs light to drive the water‐splitting reaction for hydrogen generation. The QFF layer supports the PTC layer and aids in forming the photothermal interface. The thermal conductivity property of the FAC layer creates a localized high‐temperature region at the interface rather than dispersing heat uniformly throughout the water. The underlying liquid water is continuously supplied to the photothermal interface via capillary action through the FAC layer and surface evaporation. Consequently, the photothermal synergistic water splitting process, powered solely by sunlight, achieves continuous hydrogen production given an adequate supply of liquid water. In this study, we have employed Rh, Cr, and Co‐loaded Al‐doped SrTiO_3_ as the prepared PTC, uniformly dispersed on the QFF. X‐ray diffraction (XRD) characterized the crystalline structures of SrTiO_3_, Al‐doped SrTiO_3_, and co‐catalyst‐loaded SrTiO_3_:Al. The diffraction patterns of the three catalysts closely matched reference pattern #84‐0443, indicating minimal changes in their crystalline structures (Figure 1b). This finding implies that both doping and surface loading minimally affect the catalysts’ crystal structure. Additionally, transmission electron microscopy (TEM) coupled with energy‐dispersive spectroscopy (EDS) revealed a homogeneous distribution of Ti, Sr, O, and Al elements in the SrTiO_3_ lattice, with Rh, Cr, and Co elements predominantly on the surface.^[^ ^14^ ^]^ This distribution suggests effective Al doping within the SrTiO_3_ lattice and successful loading of Rh, Cr, and Co on the crystal surface (Figure 1c). UV–vis absorption spectroscopy investigated the illumination absorption characteristics of the prepared catalyst (Figure S1, Supporting Information). The inherent absorption edge for all samples occurred at ≈380 nm. Employing Kubelka–Munk theory, the bandgaps of the samples were determined to be ≈3.27 eV, demonstrating no significant shifts with Al doping or cocatalyst loading.

FAC is a crucial component of the interfacial photothermal system. Detailed TEM‐EDS analysis shows that FAC primarily comprises Si, O, and Al elements, collectively representing over 95% of its composition (Figure 1d). Minor quantities of trace elements like Ca, K, Na, Mg, and Fe were also detected. Figure S2 (Supporting Information) provides a comparative images of untreated, washed, and calcined FAC. Initially, FAC appears grayish‐white with significant ash deposits in aqueous media. Washing results in a noticeable whitening. After calcination, FAC acquires a pink hue, likely due to complete oxidation during the process, which leads to oxide formation and enhanced color intensity. X‐ray photoelectron spectroscopy (XPS) analysis corroborates this finding by revealing an increase in trivalent iron content from 28% to 92% post‐calcination (Figure S3, Supporting Information). Since trivalent iron typically displays a dark red color, the color change in FAC can be primarily attributed to the increased presence of this iron species. The particle size of FAC was characterized, showing a micron‐scale size, predominantly ≈340 µm in diameter (Figure S4, Supporting Information). It is reported that micron‐sized pores are more suitable for efficient capillary water pumping than nanopores.^[^ ^20^ ^]^ The density of the FAC, tested at 0.234 g cm^−3^, enables the entire system to float on water. Continuous testing for up to 90 days showed that FAC maintains excellent mechanical and chemical stability, remaining afloat even as water levels decrease due to evaporation (Figure S5, Supporting Information). Whether FAC is added to water or vice versa, the floating system forms spontaneously and quickly, even in irregular containers (Figure S6, Supporting Information). Water contact angle test on FAC revealed a contact angle of 8 degrees, highlighting its exceptional hydrophilicity (Figure 1e). It can be seen that calcination enhances this hydrophilicity, facilitating efficient water transport within the system.^[^ ^21^ ^]^ Measurements of the thermal conductivity of FAC indicate a value of only 0.084 W m^−1^ K^−1^. This value is superior to some recently reported thermal insulation materials (Figure 1f). The low thermal conductivity of FAC is beneficial for maintaining the stability of the interfacial photothermal reaction system. These results underscore the unique properties and potential applications of FAC in interfacial photothermal systems, suggesting pathways for future advancements in the field.

### 2.2 Photothermal Synergistic Water Splitting on PTC/QFF/FAC System

**Figure 2:**
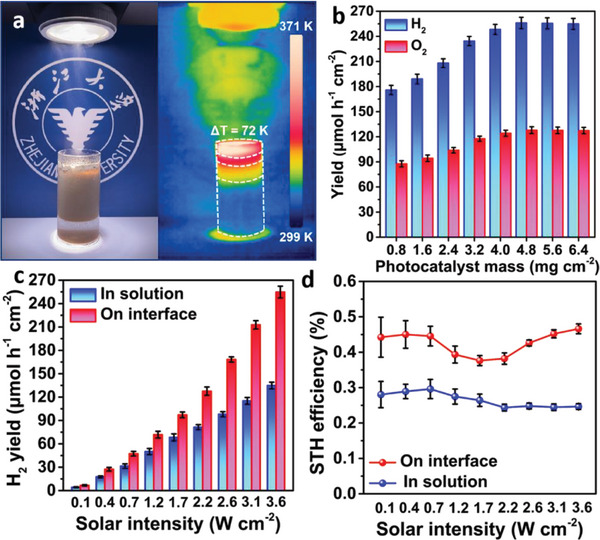
The designed PTC/QFF/FAC system under illumination testing. a) Photograph (left) and the infrared radiation thermal image (right) from PTC/QFF/FAC system under illumination. b) Mass loading‐dependent photocatalytic gas production rates for the PTC/QFF/FAC system (area: 3.14 cm^2^). Comparisons of c) H_2_ evolution rates and d) STH efficiency between the liquid water/catalyst/hydrogen three‐phase system and the PTC/QFF/FAC interfacial photothermal system (area: 3.14 cm^2^, mass: 15 mg) under different illumination intensities.

**Figure** **2**a shows the radiation thermal image of the PTC/QFF/FAC system, revealing intriguing temperature gradients. Under focused illumination, a concentrated interfacial heat zone is observed in a narrow region, ≈5 mm thick, of the upper layer. In stark contrast, the bulk liquid at the bottom maintains a temperature close to room temperature, indicating efficient thermal insulation and localization of the photothermal effect.^[^ ^22^ ^]^ These observations suggest that the unique thermal properties of FAC facilitate the establishment and maintenance of a stable interfacial photothermal reaction system, crucial for various applications in photothermal chemistry and beyond. The impact of varying PTC mass loadings on the photothermal catalytic hydrogen production rate within the PTC/QFF/FAC system was investigated (Figure 2b). As the loading amount increased, so did the hydrogen production rate, with an optimal mass loading of ≈4.8 mg cm^−^^2^ representing the optimal mass loading. Further increases in mass led to a slight decrease in hydrogen production, possibly due to the excessive thickness of the catalyst layer, which could reduce contact between the catalyst and reactant water.^[^ ^23^ ^]^ It was consistently observed that the ratio of hydrogen to oxygen maintained a stoichiometric balance of 2:1. To elucidate, the photothermal catalytic activity of the QFF/FAC system was assessed independently under identical conditions; no hydrogen or oxygen was detected after 3 h, confirming that the catalytic activity of the PTC/QFF/FAC system is attributable to the PTC.

The superiority of the PTC/QFF/FAC interfacial photothermal system was demonstrated through a comparative study with the liquid water/catalyst/hydrogen three‐phase system (Figure 2c). The experimental results revealed that under various illumination intensities, the hydrogen production rate of the PTC/QFF/FAC interfacial photothermal system surpassed that of the three‐phase system, achieving 254.82 µmol h^−1^ cm^−2^ compared to 134.75 µmol h^−1^ cm^−2^ for the three‐phase system, an 89% increase in production (at 3.6 W cm^−2^). Noteworthy, with increasing illumination intensity, significant changes were observed in STH: the three‐phase system showed a distinct downward trend in STH after reaching a peak (Figure 2d). The STH of the PTC/QFF/FAC system initially increased, then decreased, and later rose substantially once the illumination intensity surpassed ≈1.7 W cm^−2^, even exceeding lower light intensities. STH reached 0.47% under the illumination intensity of 3.6 W cm^−2^, which is 0.02% higher than 0.45% under 0.4 W cm^−2^. This further suggests that the PTC/QFF/FAC system exhibits a more pronounced advantage in hydrogen production when operated under high illumination intensities. To assess the system's stability, repeated photocatalytic synergistic water splitting experiments for hydrogen production were conducted. Over ten cycles under identical conditions, the hydrogen production rate averaged 238.3 µmol h^−1^ cm^−2^, demonstrating the stable performance of the PTC/QFF/FAC system (Figure S7, Supporting Information). The observed decline in hydrogen yield is primarily attributed to the loss of PTC during the recycling processes.

### 2.3 Mechanism of Photothermal‐Interface Effect on Catalytic Performance

To elucidate the superior hydrogen evolution performance of the PTC/QFF/FAC interfacial photothermal system compared to the water/catalyst/hydrogen three‐phase system, a comprehensive series of experiments and characterizations were performed. Hydrogen production rates for the three‐phase reaction were assessed at various temperatures under consistent light intensity using a 365 nm LED light source, with temperatures elevated through external heating. As depicted in Figure S8 (Supporting Information), a continuous increase in hydrogen production rate was observed with increasing reaction temperatures. At 370 K, the hydrogen production rate reached 180.69 µmol h^−1^ cm^−2^, marking a 95.44% increase from 92.45 µmol h^−1^ cm^−2^ at room temperature. Additionally, the relationship between the H_2_ evolution rate (*V*) and the reaction temperature (*T*) was modeled using the Arrhenius equation:^[^ ^24^ ^]^

(1) $$V=3277.52e^{-\left(\frac{8919.18}{8.314*T}\right)}$$

**Figure 3:**
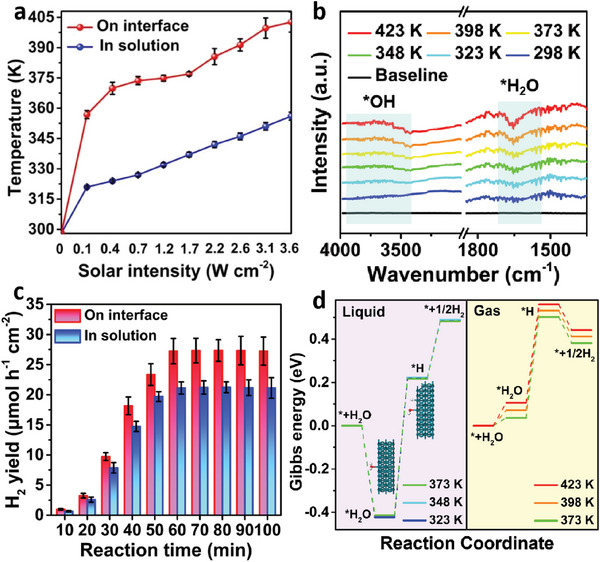
Investigation of the photothermal‐interface effect on catalytic performance. a) Variation of reaction temperatures in the PTC/QFF/FAC system and the three‐phase system with changes in light intensity. b) FTIR spectroscopy at different temperatures. c) Comparison of catalytic water splitting performance between the PTC/QFF/FAC interface photothermal system and the three‐phase system at the same light intensity (0.4 W cm^−2^) and temperature (370 K). d) Gibbs energy of a photocatalytic reaction in the liquid (left) and gas(right) system at different temperatures.

The activation energy required for hydrogen production was determined to be 8.919 kJ mol^−1^ (Figure S9, Supporting Information). These results indicate that temperature elevation is a key factor enhancing the hydrogen evolution reaction. The temperature curves provide insights into the trends in solar‐to‐hydrogen (STH) efficiency (**Figure** **3**a). The region of decreasing STH efficiency in the interfacial photothermal reaction corresponds to the latent heat associated with the water phase transition.^[^ ^25^ ^]^ In this phase, despite an increase in energy input, the reaction temperature remains relatively constant, resulting in a decreased STH efficiency.^[^ ^26^ ^]^ In the three‐phase reaction, the temperature linearly increases with rising light intensity, while STH efficiency initially increases and then decreases. This behavior is attributable to moderate temperature increases promoting the forward reaction, thus enhancing hydrogen evolution.^[^ ^27^ ^]^ However, there are some studies have indicated that elevated temperatures can promote the reverse reaction, specifically the recombination of hydrogen and oxygen.^[^ ^28^ ^]^ Additionally, photocatalytic reactions in liquid water result in the loss of cocatalyst, thus reducing STH efficiency. To further elucidate the factors influencing the enhanced hydrogen production rate in the PTC/QFF/FAC system under intense light conditions, temperature‐dependent UV absorption measurements were conducted (Figure S10, Supporting Information). The results demonstrated an enhancement in the absorbance of the photothermal catalyst as the temperature increased. According to the Kubelka–Munk theory, the bandgap narrowed from 3.25 eV (323 K) to 3.22 eV (423 K). This narrowing can be attributed to increased electron‐phonon interactions at elevated temperatures, which enhance lattice vibrations and expansion.^[^ ^29^ ^]^ These structural changes in the semiconductor material lead to a reduced bandgap, thereby generating a higher density of photogenerated carriers.^[^ ^30^ ^]^ Complementing these findings, temperature‐dependent photoluminescence (PL) spectra were conducted (Figure S11, Supporting Information). These plots revealed that the luminescence intensity of the photothermal catalyst gradually diminishes as the temperature rises, indicating that elevated temperatures suppress the radiative recombination of photogenerated carriers within the sample. Consequently, at higher temperatures, photogenerated carriers migrate more efficiently to the cocatalyst sites on the surface of the catalyst, which suppresses electron‐hole recombination. Concurrently, time‐resolved PL measurements across various temperatures showed a continuous decrease in the lifetime of carriers in PTC as the temperature increases, dropping from 2.30 ns at 323 K to 1.81 ns at 423 K, representing a 21.3% reduction. A shorter carrier lifetime suggests an increased carrier migration rate within the material, enabling them to reach the active sites required for catalytic reactions more rapidly, thereby facilitating participation in the reaction process. The enhancement in charge separation and migration efficiency boosts the photocatalytic activity of the PTC/QFF/FAC system, thereby enhancing the hydrogen yield.^[^ ^31^ ^]^ Temperature‐dependent Fourier transform infrared (FTIR) spectroscopy was performed, as depicted in Figure 3b. The peak observed ≈1650 cm^−1^ corresponds to the physical adsorption of undissociated water on the catalyst surface, whereas the peak ≈3800 cm^−1^ represents the adsorption of hydroxyl groups resulting from water dissociation.^[^ ^32^ ^]^ As the temperature rises, a notable decrease in the water infrared signal and a corresponding increase in the hydroxyl peak are observed. This indicates that the elevation in temperature facilitates the transition of physically adsorbed water to chemically adsorbed states, promoting faster dissociation and hydroxyl production.^[^ ^33^ ^]^ Therefore, the elevation of temperature is advantageous for water splitting.

To explore the promotional effects beyond temperature, both systems were assessed for hydrogen evolution under the same temperature and light intensity conditions, as depicted in Figure 3c. The hydrogen evolution rate under the interfacial photothermal reaction was consistently higher than that of the three‐phase reaction, even at identical temperature conditions, as the reaction progressed. After 80 min of illumination, the interfacial photothermal system reached 27.34 µmol h^−1^ cm^−2^, which is 28.66% higher than 21.25 µmol h^−1^ cm^−2^ observed in the three‐phase reaction. This indicates that the PTC/QFF/FAC system inherently offers a superior hydrogen evolution advantage over the three‐phase reaction, attributable to the enhanced hydrogen transfer capability afforded by the photothermal interface reaction environment. To further elucidate, Density Functional Theory (DFT) calculations were employed at various temperatures in both liquid and gas phases to simulate the adsorption of *H_2_O molecules, hydrogen atoms (*H), and the generation of H_2_ on the Rh (111) facet (Figure S12, Supporting Information). The results are presented in Figure 3d. Based on these Gibbs free energy calculations, it is evident that the decomposition of *H_2_O into *H represents the rate‐determining step of the reaction. In the liquid phase, the energy required for the transition from *H_2_O to *H decreases from 0.6426 eV at 323 K to 0.6312 eV at 373 K, and in the gas phase, it decreases from 0.4656 eV at 373 K to 0.4539 eV at 423 K. This underscores temperature as a critical factor influencing the catalytic process, where an increase in temperature facilitates the decomposition of water into hydrogen protons and hydroxyl groups. Furthermore, at the same temperature of 373 K, the energy required for the transition from *H_2_O to *H in the gas‐phase system is 0.4656 eV, lower by 0.1656 eV than the 0.6312 eV required in the liquid phase. This indicates that the interfacial photothermal catalytic system is better suited for water splitting reactions, and the phase transition is key to enhancing the overall performance of the catalytic system.

Additionally, hydrogen gas diffusion coefficients in various systems were estimated using liquid‐phase and gas‐phase diffusion equations, illustrating the impact of phase states on hydrogen evolution performance. In the liquid phase, the hydrogen gas diffusion coefficient (*D*_L_, m^2^ s^−1^) was using the Stokes–Einstein equation:^[^ ^34^ ^]^

(2) $$D_{L}=7.4*10^{-8}\frac{{\left(\varphi_{H_{2}O}M_{H_{2}O}\right)}^{0.5}T}{\mu V_{H_{2}}^{0.6}}$$

where *T* represents the temperature (K), $M_{H_{2}O}$ is the molecular mass of water, $\varphi_{H_{2}O}$ is the association parameter of water with a value of 2.6, and *µ* denote the viscosity of water, respectively, and $V_{H_{2}}$ is the molar volume of hydrogen.

The hydrogen gas diffusion coefficient (*D*_G_, m^2^ s^−1^) in the gas phase can be calculated by the Chapman–Enskog theory:^[^ ^35^ ^]^

(3) $$D_{G}=1.858*10^{-3}\frac{T^{\frac{3}{2}}\sqrt{\frac{1}{M_{H_{2}}}+\frac{1}{M_{H_{2}O}}}}{P*\Omega *\sigma^{2}}$$

where *M* is the molar mass, *P* is the system pressure, *σ* is the average collision diameter of the molecule, and Ω is a temperature‐dependent collision integral. With an increase in temperature, both *D*_L_ and *D*_G_, the hydrogen gas diffusion coefficients, are enhanced. Consequently, hydrogen transport resistance is reduced, and higher temperatures facilitate more efficient gas transport and diffusion. Due to interfacial frictional resistance, the *D*_L_ and *D*_G_ differ significantly even at identical temperatures. At ≈373 K, the calculated hydrogen diffusion coefficient *D*_G_ is two orders of magnitude higher than *D*_L_, which are 2.65 × 10^−3^ and 5.06 × 10^−5^ m^2^ s^−1^, respectively. This suggests that the hydrogen evolution reaction under interfacial photothermal conditions is more advantageous than that in the three‐phase system. Moreover, temperature‐programmed desorption (TPD) tests revealed that an increase in temperature enhances hydrogen desorption (Figure S13, Supporting Information).

Based on these observations, it is evident that the interfacial photothermal system enhances water splitting, particularly under high light intensities. First, the photothermal interface concentrates heat within a thin layer, minimizing heat dissipation and elevating reaction temperatures. This enhances the light absorption and photogenerated carriers transfer capabilities of the photothermal catalyst, concurrently reducing the activation energy necessary for water decomposition. Second, the photothermal interface facilitates phase transition processes, thereby expediting gas product separation, mitigating reverse reactions, and fostering water decomposition for hydrogen generation, as compared to traditional three‐phase reaction systems.

### 2.4 Photothermal Synergistic Water Splitting in Scaled‐Up Experiments

**Figure 4:**
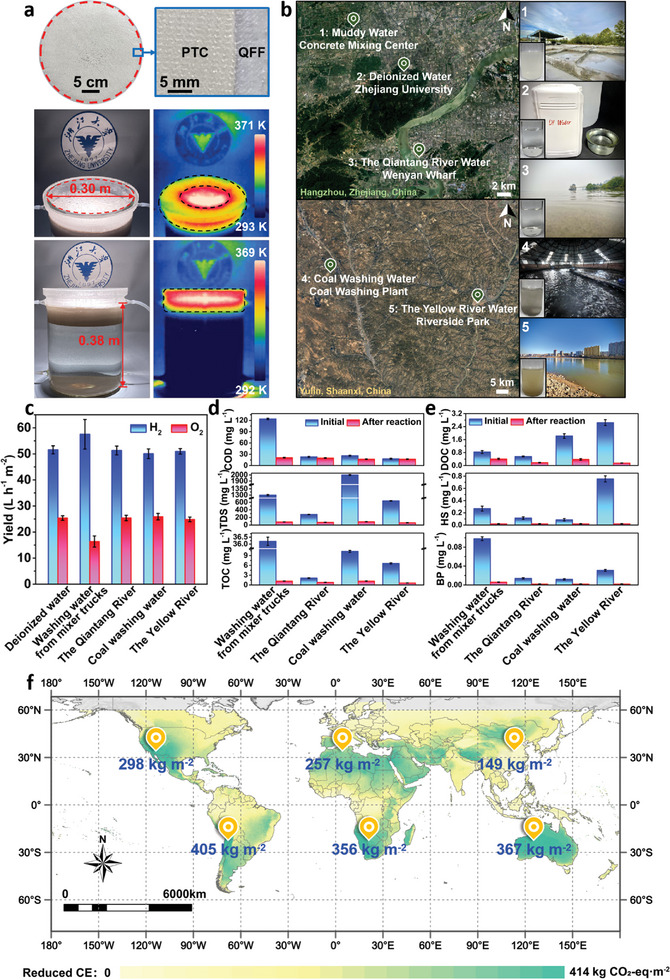
Concurrent hydrogen production and water purification in the designed expanded system. a) Images of the expanded PTC/QFF. The corresponding SEM and EDS mapping images are provided below. b) Sampling map of various water sources across different regions. c) Gas generation rates of five different water under illumination (3.2 W cm^−2^). d) Physical and chemical properties of four different water before and after purification. e) Variations in organic matter content in four waters before and after the reaction. f) Carbon emission reduction map derived from the hydrogen production data of the scaled‐up system (incorporating global annual light exposure parameters).

To further validate the applicability of the PTC/QFF/FAC system, expanded experiments were conducted. A quartz container with dimensions of 0.3 × 0.3 m^2^, featuring inlet and outlet ports at the top, was designed. As shown in **Figure** **4**a, PTC is laid flat on the QFF surface, forming a thin film with an average thickness of ≈120 µm. Under illumination, this system rapidly generates localized high temperatures, concurrently producing large quantities of hydrogen and purified water (Figure 4b). Various natural waters were used to assess the scalability of the system's design, including river water from the Yellow River and the Qiantang River, along with industrial muddy waters from mixer trucks and coal washing processes. The corresponding water quality parameters are provided in Table S1 (Supporting Information). Wastewater from mixer trucks displays a milky white hue and high turbidity, whereas coal washing water is gray, containing black coal particles of varying sizes. Water from the Yellow River appears muddy and yellowish‐brown, enriched with dark‐colored sand and gravel. These particles significantly absorb light, thereby reducing the catalyst's light absorption capacity. Nevertheless, the PTC/QFF/FAC system confines catalysis to a thin surface layer, effectively mitigating the impact of particles on light absorption.

Under xenon lamp irradiation, the hydrogen production yield were obtained as shown in Figure 4c. Photothermal synergistic water splitting experiments conducted across various waters consistently yielded hydrogen gas rates exceeding 50 L h^−1^. In deionized water, the experiment achieved a hydrogen production rate of 51.56 L h^−1^. For river water, the H_2_ evolution rates were 51.29 L h^−1^ in the Qiantang River water and 50.94 L h^−1^ in the Yellow River water. These results suggest that organic matter and other contaminants in river water negligibly affect the performance of photothermal catalysis, with the system effectively confining catalysis at the interface. For turbid industrial wastewater, the H_2_ production rates were 57.51 L h^−1^ from mixer truck washing water and 50.05 L h^−1^ from coal washing water, further demonstrating the device's ability to circumvent light absorption and scattering by the liquid feedstock, thereby allowing direct access to the catalyst surface and achieving efficient photothermal synergistic catalytic water splitting. Furthermore, an increased hydrogen production rate and a reduced oxygen yield in mixer truck washing water suggest that organic contaminants may act as sacrificial agents, enhancing hydrogen evolution and concurrently consuming oxygen, thus purifying the hydrogen gas produced.^[^ ^36^ ^]^ This finding has significant implications for the scalability of hydrogen production and storage applications. This system has great potential for simultaneously large‐scale hydrogen production and water purification under light illumination. Water quality analysis before and after the reaction indicated significant purification of freshwater by the scaled‐up system. The analysis of chemical oxygen demand (COD) and total organic carbon (TOC) assays unveiled a significant presence of organic matter within mixer truck washing water,^[^ ^37^ ^]^ thereby fostering the generation of hydrogen gas and substantial consumption of oxygen during the photothermal catalytic process (Figure 4d). According to the World Health Organization's (WHO) Guidelines for Drinking‐water Quality, water with a total dissolved solid (TDS) level below 600 mg L^−1^ is considered palatable. The TDS values of the four purified samples are all significantly lower than 600 mg L^−1^. Additionally, parameters such as pH, total hardness (TH), total salts (TS), total phosphorus (TP), total nitrogen (TN), and total suspended solids (TSS) of the four purified waters were tested (Figure S14, Supporting Information). The specific water quality parameters after reaction are shown in Table S2 (Supporting Information). Post‐reaction, the water was purified by the interfacial photothermal reaction, potentially rendering it suitable for direct use as drinking water. Particularly for natural waters, detecting organic matter content is crucial. As shown in Figure 4e, reductions in dissolved organic carbon (DOC), humic substances (HS), and bio‐polymers (BP) were observed in the four water sources. After purification, their values decrease notably. These results demonstrate that both organic and inorganic contents in the water decrease following the interfacial photothermal reaction, underscoring the effective purification achieved. The performance of the PTC/QFF/FAC system was consistently high across all tested water types, further validating the approach's versatility. To further demonstrate the superiority of the system, we calculated the carbon emission reduction achievements worldwide by integrating global solar energy resource data from SolarGIS Ltd. As shown in Figure 4f, it was determined that our interfacial photothermal system could achieve a carbon dioxide emission reduction of 414 kg m^−2^, potentially having a profound positive impact on the global environment and climate. Based on the current global map of large‐scale photovoltaic solar energy generating units,^[^ ^38^ ^]^ we selected six locations and annotated the calculated results. It can be observed that the carbon dioxide emission reduction benefits are notably significant around Southern Peru and Western Australia, reaching 405 and 367 kg m^−2^, respectively.

## 3 Discussion

In summary, we present a novel PTC/QFF/FAC interfacial photothermal reaction system, utilizing cost‐effective industrial byproduct fly ash cenospheres, capable of withstanding high temperatures and intense light. This system achieved a remarkable hydrogen production rate of 254.8 µmol h^−1^ cm^−2^, marking an 89% enhancement over conventional three‐phase systems. Through comprehensive experimental tests and simulation calculations, we elucidated the underlying mechanism of water splitting. The findings reveal that the photothermal interface efficiently concentrates heat within a thin layer, minimizing heat dissipation and elevating reaction temperatures, thus enhancing light absorption and carrier transfer capabilities of the photothermal catalyst, and concurrently reducing the activation energy required for water decomposition. And the photothermal interface facilitates phase transition processes, expediting gas product separation, mitigating reverse reactions, and promoting hydrogen generation from water splitting. Furthermore, we have successfully developed a scaled‐up reaction system, demonstrating its capability for large‐scale hydrogen production and simultaneous water purification, achieving hydrogen yields exceeding 50 L h^−1^ m^−2^ across various water sources. Carbon emission analysis underscores the environmentally sustainable nature of our system. This study proposes a promising approach harnessing high solar irradiance for wastewater treatment, hydrogen production, and clean water generation, offering significant implications for future energy‐efficient and sustainable practices.

## 4 Experimental Section

### Preparation of FAC

The preparation protocol for FAC consists of two principal phases. Phase 1: the FAC were initially deposited in a beaker, where they undergo multiple wash cycles with deionized water until the runoff was clear. Each cycle includes a 15 min ultrasonic treatment followed by a 5 min settlement period. Post‐rinsing, FAC was transferred to a new beaker, carefully leaving behind the sedimented impurities. This step was repeated, substituting deionized water with anhydrous ethanol for three final washes. Phase 2: The purified FAC was positioned in a crucible and subjected to a 10 h calcination at 1173 K within a muffle furnace. A color transition from off‐white to light pink post‐calcination visually confirms the FAC’ readiness.

### Preparation of SrTiO_3_:Al Photocatalysts

SrTiO_3_ doped with Al (SrTiO_3_:Al) was synthesized via a molten‐salt‐mediated approach using commercially available precursors. The precursors, SrCl_2_, Al_2_O_3_, and SrTiO_3_, were combined in a molar ratio of 10:0.02:1 and homogenized in an agate mortar. The resultant mixture was then transferred to a high‐purity alumina crucible and subjected to a temperature of 1423 K in an ambient air atmosphere for 10 h. After cooling to ambient temperature, the product underwent five washing cycles with distilled water until the supernatant pH was neutral, ensuring the removal of any residual reactants or byproducts.

### Cocatalyst Loading on SrTiO_3_:Al

Cocatalysts were loaded onto SrTiO_3_:Al photocatalysts via photodeposition. Initially, photocatalysts were dispersed in deionized water. Aqueous solutions of RhCl_3_·6H_2_O, K_2_CrO_4_, and Co(NO_3_)_2_·6H_2_O were prepared and subsequently irradiated under a Xe lamp (300 W, λ > 250 nm; Profect Light) to facilitate deposition. Cocatalyst loadings were set at 0.1 wt% Rh, 0.05 wt% Cr, and 0.05 wt% Co. Each solution was added sequentially to the suspension and exposed to light for durations of 15 min for Rh, followed by 7.5 min each for Cr and Co.

### Preparation of PTC/QFF

The quartz fiber filter (QFF) was preconditioned by immersing it in anhydrous ethanol, followed by a 15 min sonication. The QFF was then thermally treated in a crucible at 873 K for 6 h and cooled to ambient temperature. The treated QFF was placed on a layer of flat absorbent cotton, with a 20 mm × 20 mm quartz ring precisely centered on top. Subsequently, 15 mg of PTC dissolved in 5 mL of water was shaken for 1 min, then poured onto the quartz ring, allowing rapid absorption by the underlying cotton and forming a catalyst layer on the QFF, completing the preparation of PTC/QFF.

### Assembly of the PTC/QFF/FAC System

The assembly process began by adding 15 g of FAC to 50 mL of deionized water in a quartz cup, with vigorous shaking to release trapped air until no visible bubbles remain in the FAC layer, while maintaining a level surface on the upper layer. The prepared PTC/QFF was then horizontally placed on top of the FAC layer, with slight shaking to ensure optimal contact at the interface. The system was finalized by the formation of a thin water film on the PTC, signifying the successful construction of the PTC/QFF/FAC system.

### Characterization

Structural characterization was performed using X‐ray diffraction (XRD) with a Rigaku SmartLab SE diffractometer. Sample morphology was assessed via scanning electron microscopy (SEM) on a ZEISS Sigma 300 and transmission electron microscopy (TEM) on an FEI Talos F200S. Surface chemistry was probed using X‐ray photoelectron spectroscopy (XPS) on a Thermo Scientific K‐Alpha spectrometer employing Al Kα radiation (1486.6 eV). Optical properties were evaluated using UV–vis diffuse reflectance spectroscopy (UV–vis DRS) across a wavelength range of 200–1400 nm, utilizing Shimadzu UV‐2600 and UV‐3600 iplus spectrophotometers. Particle size distribution was determined with a Malvern Mastersizer 2000. Photoluminescence (PL) emission spectra were recorded on an Edinburgh FLS980 spectrometer at an excitation wavelength of 365 nm to analyze the recombination dynamics of the photogenerated carriers. Powder samples weighing 20 mg were used for these measurements. Density was measured using the AccuPyc II 1340 from Micromeritics. Surface wettability was assessed by contact angle measurements using a Dataphysics DCAT21. Spread ≈200 mg of FAC evenly on the measurement platform. Deposit the liquid using instrument‐controlled dispensing. Observe and record the liquid's behavior on the surface with high‐precision cameras and sensors to determine the dynamic water contact angle. Repeat the process three times and calculate the average contact angle. Thermal conductivity was determined using a Hot Disk TPS2500S. Fix the measurement probe to the sample holder, place the FAC in the mold, and secure the probe to ensure full contact with the FAC sample. Conduct the test to measure thermal conductivity, repeating the process three times and calculating the average value. These methods elucidated the samples’ physical and chemical attributes, essential for evaluating their photocatalytic efficacy.

### Hydrogen Generation Tests

Hydrogen production in the PTC/QFF/FAC system was quantified using a stainless steel reactor equipped with a sapphire window. The chamber was positioned 12 cm from a 300 W xenon lamp, with spectral output continuously monitored. Exiting gases were measured using an Alicat flowmeter and analyzed via gas chromatography on an Agilent 8020A system employing an argon carrier and a molecular sieve‐5A column. Measurements were taken every 10 min.

For the stability testing of the PTC/QFF/FAC system, the process was repeated with each cycle allowing the reaction to proceed until the hydrogen production rate stabilized (≈3 h). The average hydrogen yield from three consecutive stable periods was taken as the data for a single cycle. A new cycle commenced with the same PTC/QFF/FAC system, refilling deionized water to the initial level post each cycle, while maintaining all other conditions consistent.

For expanded trials, the apparatus was contained within a sealed quartz reactor, with gas flows regulated by a flowmeter and compositions determined by gas chromatography.

Another set of experiments was conducted in a liquid water/photocatalyst/hydrogen three‐phase system using a quartz cup containing 10 mL of deionized water. PTC powder was added to the quartz cup and thoroughly stirred to ensure its complete dispersion in the water, forming an opaque, turbid liquid. Throughout this process, the illumination area, distance, and catalyst mass were kept consistent with the main system. Hydrogen evolution from water splitting was then tested. Temperature during all tests was monitored with a thermocouple.

### Theoretical Calculation

The STH (solar‐to‐hydrogen) efficiency refers to the ratio of solar energy stored as H_2_ to the incident solar energy and is one of the most critical factors for the practical implementation of photocatalytic OWS (overall water spitting) for solar hydrogen production. The STH efficiency was calculated according to the following formula:^[^ ^39^ ^]^

(4) $$\mathrm{STH}\left(\%\right)=\frac{Output\;energy\;as\;H_{2}}{Energy\;of\;incident\;solar\;light}=\frac{r_{H_{2}}*\Delta G}{P*A}\;*100\%$$

where $r_{H_{2}}$ represents the hydrogen evolution rate of H_2_, Δ*G* is the Gibbs free energy of the reaction (taken as 237 kJ mol^−1^), *P* denotes the energy flux of incident sunlight, and *A* is the photoactive area of the device.

First‐principles calculations were performed using the CP2K Quickstep package.^[^ ^40^ ^]^ Calculations were performed within DFT formalism based on a hybrid Gaussian plane wave scheme using the PBE‐GGA exchange correlation function.^[^ ^41^ ^]^ The empirical correction in DFT‐D3 method with Becke‐Jonson damping was employed to describe the van der Waals interactions. The norm‐conserving GTH pseudopotentials were used to describe the core electrons.^[^ ^42^ ^]^ The basis sets for the valence electrons (1s 1 for H, 2s 2 2p4 for O and 4s 2 4p6 4d8 5s 1 for Rh) were treated by short‐ranged (less diffuse) and double‐z basis functions with one set of polarization functions (DZVP).^[^ ^43^ ^]^ The cut‐off energy of an auxiliary basis set of plane waves was 500 Ry. All of the atomic positions were fully relaxed until the force was smaller than 4.5×10^−4^ a.u./bohr. The convergence threshold for the self‐consistent‐ field iteration was set to 5 × 10^−6^ a.u. The convergence accuracy of energy was set to 1×10^−11^ a.u. To aviod the interaction between adjacent slabs, a vacuum layer thickness of 15 Å is chosen along the surface normal direction.

In order to compare the performance of photocatalytic hydrogen evolution at different temperatures in the liquid and gas phases, the SCCS Implicit solvent model was employed to conduct structural relaxation and vibration analysis for each intermediate. The dielectric constants of liquid water and gas water are indexed to be 78.36 and 1, respectively. Based on the thermodynamic formula (G = E+ZPE+PV‐TS), the Shermo program^[^ ^44^ ^]^ was used to calculate the Gibbs free energy of key intermediates under specific conditions (p = 1 bar, T = 323.15, 3.48.15, 373.15, 398.15, and 423.15 K).

The global carbon emission reduction (*R*_CE_) were calculated using the global direct normal irradiation (DNI). DNI is the most critical parameter for calculating electricity output and performance evaluation of concentrated solar power and concentrated photovoltaic technologies. It is also crucial for calculating the global radiation received by tilted or sun‐tracking photovoltaic modules. DNI represents the long‐term average annual/daily total of direct normal irradiance. The fundamental solar resource database was computed by the Solargis model based on atmospheric and satellite data, with a time step of 10, 15, or 30 min (depending on the region). The spatial resolution of terrain effects was 250 meters. The actual power generation from hydrogen was calculated using the higher heating value of hydrogen, combined with fuel cell power generation conversion. This allowed us to calculate the carbon dioxide emissions reduction associated with saving one kilowatt‐hour of electricity, leading to the determination of global carbon emission reduction data. The global carbon emission reduction outcomes are calculated through the following formula:

(5) $$R_{\mathrm{CE}}=365.25*10^{-3}\frac{\gamma_{H_{2}}*I_{\mathrm{GIS}}*A*q_{H_{2}}*\eta_{E}*\eta_{eq}}{M_{H_{2}}}$$

Where $\gamma_{H_{2}}$ represents the hydrogen yield; *I*_GIS_ is the GIS data of global solar irradiation; $q_{H_{2}}$ is the calorific value of hydrogen combustion (taken as 285.8 kJ mol^−1^); η_E_ is the efficiency of hydrogen power generation; η_*eq*_ is the equivalent emission reduction rate of CO_2_.

## Conflict of Interest

The authors declare no conflict of interest.

## Author Contributions

Y.X. designed and conducted the experiments, analyzed the data, and wrote the manuscript; Y.L. carried out DFT calculations; E.Z., Z.C., and G.D. conducted a part of experiments and analysis; X.Z. conducted a part of catalyst synthesis; Y.G. performed a part of experimental data analysis; Y.Z. and C.X. received the funding for the projects and supervised the whole project.

## Supporting information



Supporting Information

## Data Availability

The data that support the findings of this study are available in the supplementary material of this article.
