# Supplementary material for: Photothermal Synergistic Hydrogen Production via a Fly‐Ash‐made Interfacial Vaporific System
Source: Adv Sci (Weinh). 2024 Nov 28;12(3):2410201. doi: 10.1002/advs.202410201 (PMC11744568; doi:10.1002/advs.202410201)
Supplement: Supplementary file 1 — Supporting Information [file ADVS-12-2410201-s001.docx]

**Supplementary Information (SI)**

**Photothermal Synergistic Hydrogen Production via a Fly-Ash-made Interfacial** **Vaporific System**

Yin Xie^1^, Chenyu Xu^1,*^, Yan Liu^1^, Entao Zhang^1^, Ziying Chen^1^, Xiaopeng Zhan^1^, Guangyu Deng^1^, Yuan Gao^1^, Yanwei Zhang^1,*^

*^1^State Key Laboratory of Clean Energy Utilization, Zhejiang University, Hangzhou 310027, China*

mrxcy@zju.edu.cn (C. X), zhangyw@zju.edu.cn (Y. Z)


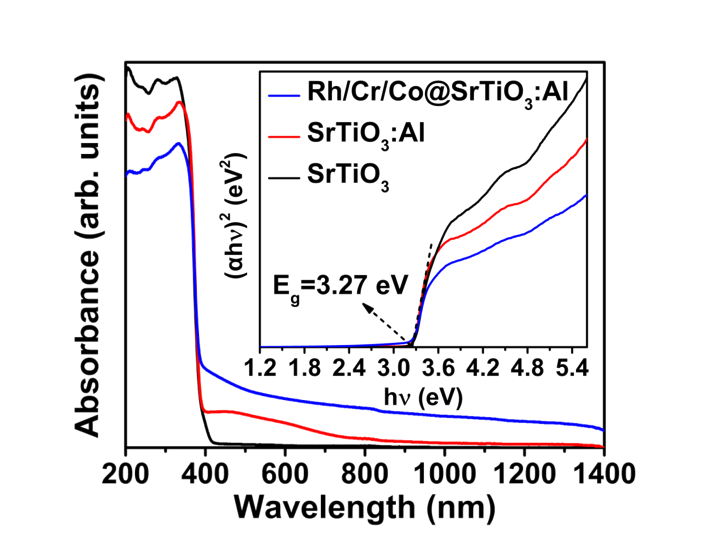


**Figure S1** UV-VIS absorption spectra and determination of the optical band gap over as-prepared catalysts (inset).


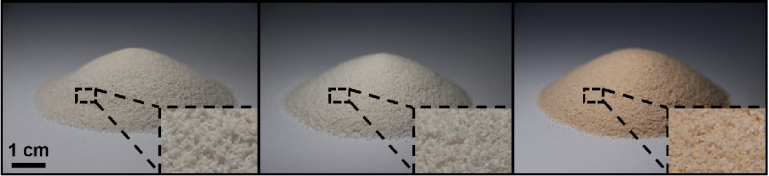


**Figure S2** The photos of original FAC (left), FAC after washing (middle), and FAC after calcination (right).


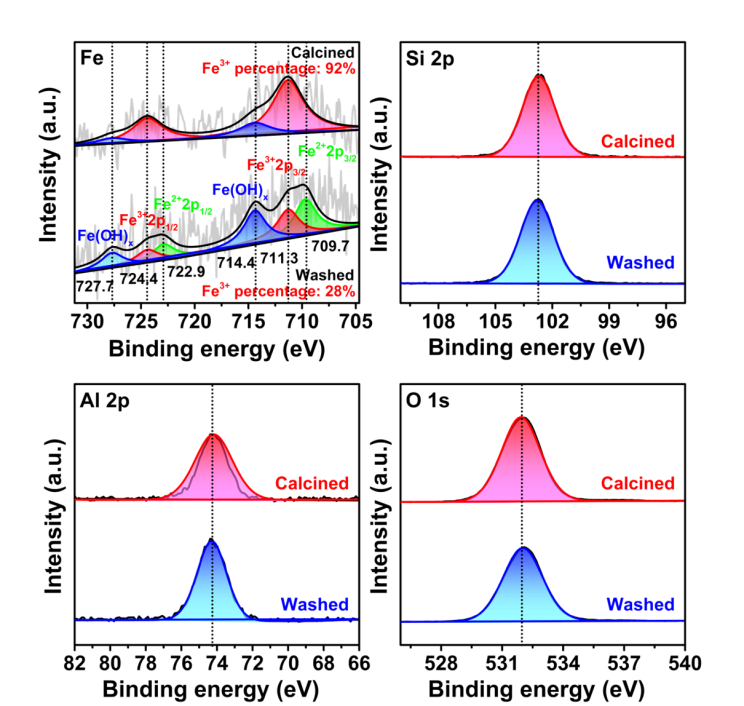


**Figure S3** XPS spectra of FAC after calcination (upper) and washing (lower)^[1]^.


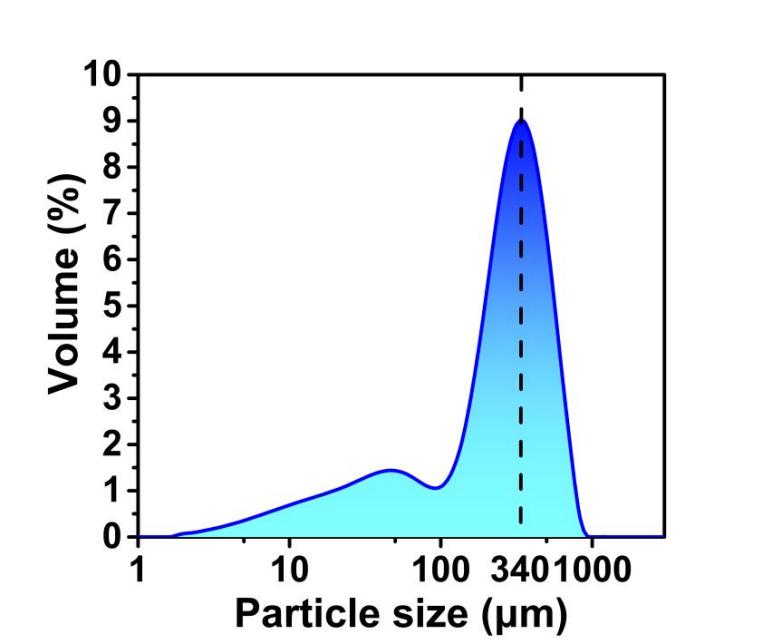


**Figure S4** The particle size distribution of FAC.


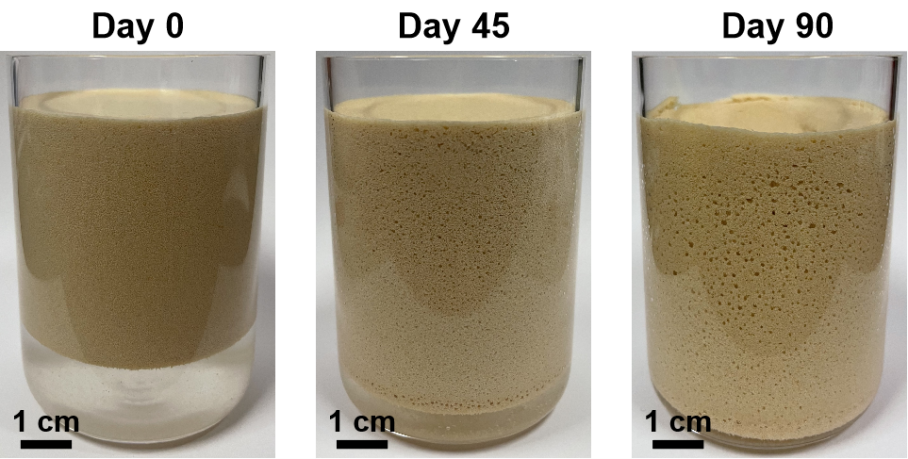


**Figure S5** Photographs of the state of 50g FAC placed in 50mL of water on day 0 (left), day 45 (middle), and day 90 (right).


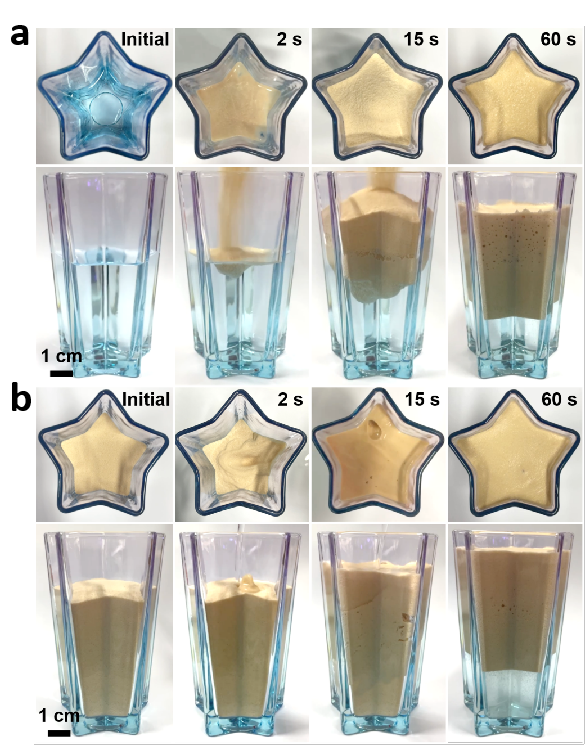


**Figure S6 a** Photos of the process of pouring FAC into water and its change over time . **b** Photos of the process of adding water to FAC and its subsequent change over time (top view above, front view below).


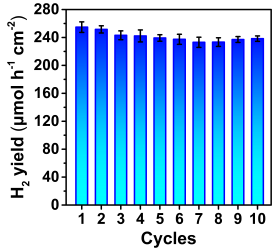


**Figure S7** Stability test of PTC/QFF/FAC system under concentrated light of 3.6 W cm^-2^. Each cycle is three hours.


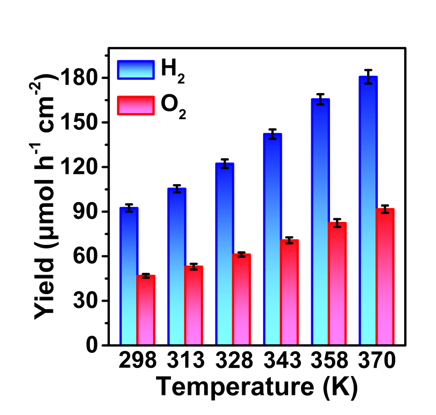


**Figure S8** Evaluation of photocatalytic water splitting performance of temperature-dependent three-phase system.


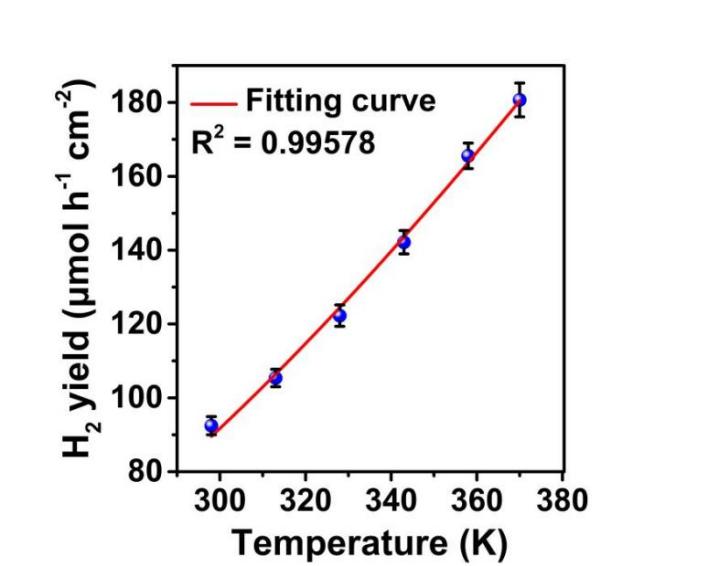


**Figure S9** The Arrhenius formula is fitted according to the hydrogen production in the three-phase reaction system at different temperatures.


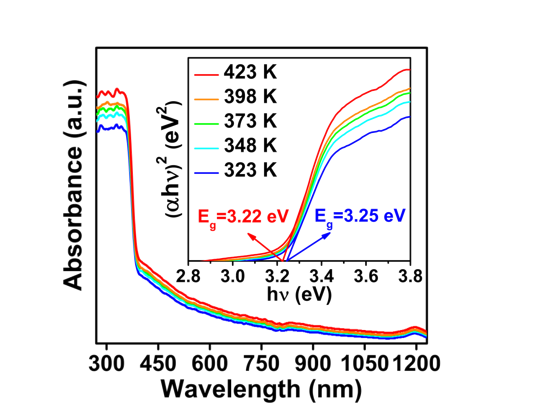


**Figure S10** Temperature-dependent UV-VIS absorption spectroscopy on PTC (Inset: temperature-dependent Tauc plots).


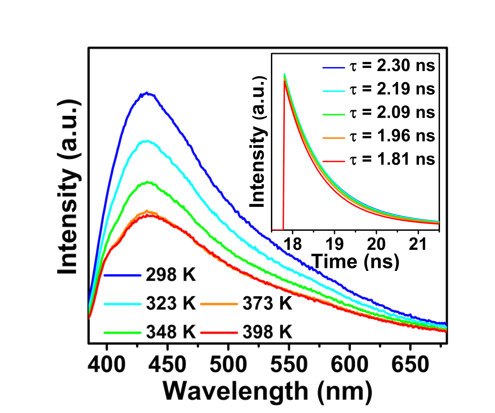


**Figure S11** Temperature-dependent PL spectra (Inset: time-resolved PL spectra plots at different temperatures).

**
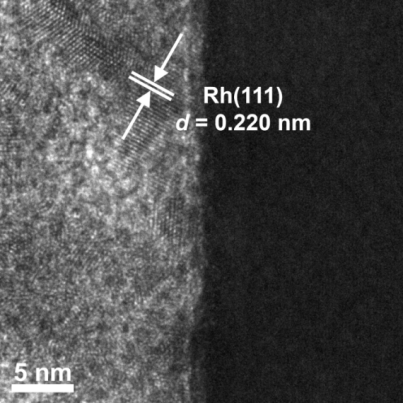
**

**Figure S12** HRTEM of the supported Rh cocatalyst on the SrTiO_3_:Al.

**
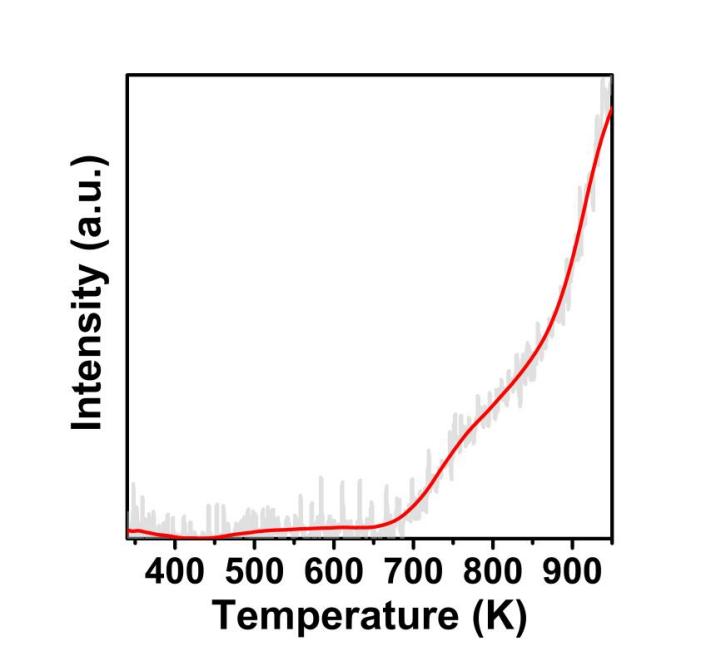
**

**Figure S13** The TPD test of H_2_ on the Rh/Cr/Co@SrTiO_3_:Al photothermal catalyst.

**
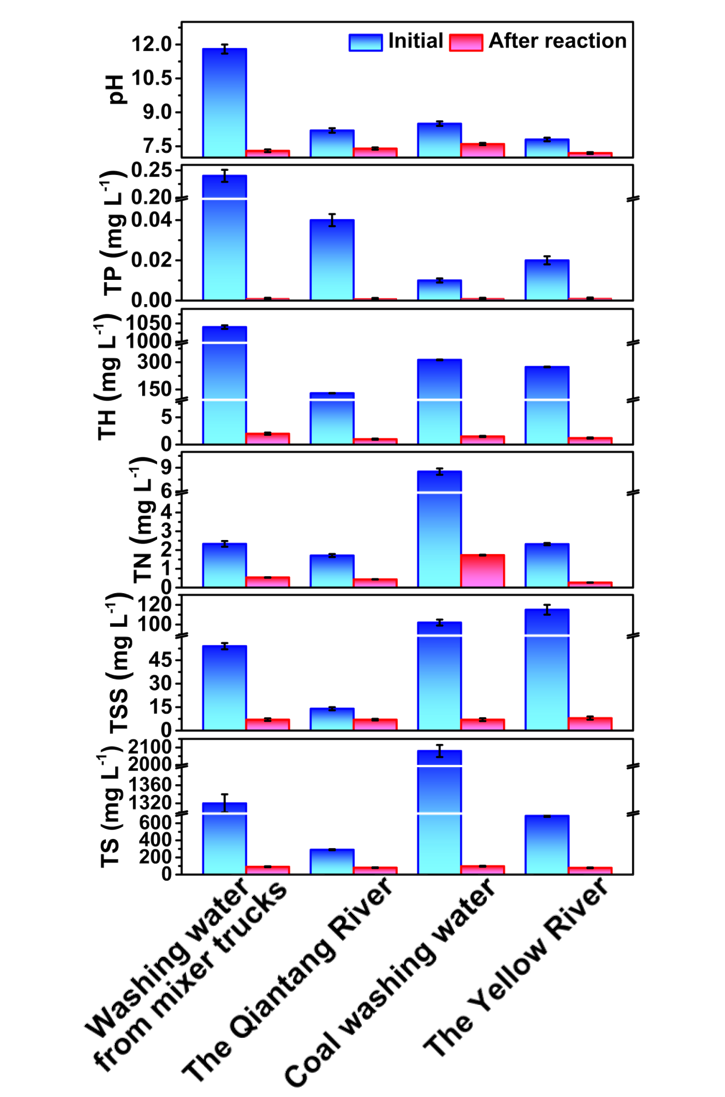
**

**Figure S14** Physical and chemical properties of four different water before and after purification.

**Table S1** Water quality parameters of the original four kinds of water.

|  | Washing water from mixer trucks | The Qiantang River | Coal washing water | The Yellow River |
| --- | --- | --- | --- | --- |
| COD (mg L^-1^) | 124 | 23 | 26 | 18 |
| TDS (mg L^-1^) | 1300 | 278 | 2000 | 638 |
| TOC (mg L^-1^) | 36.2 | 2.1 | 10.3 | 6.6 |
| DOC (mg L^-1^) | 0.833 | 0.56 | 1.82 | 2.648 |
| HS (mg L^-1^) | 0.269 | 0.114 | 0.086 | 0.756 |
| BP (mg L^-1^) | 0.098 | 0.014 | 0.012 | 0.031 |
| pH | 11.8 | 8.2 | 8.5 | 7.8 |
| TP (mg L^-1^) | 0.24 | 0.04 | 0.01 | 0.02 |
| TH (mg L^-1^) | 1040 | 130 | 313 | 274 |
| TN (mg L^-1^) | 2.33 | 1.71 | 8.51 | 2.32 |
| TSS (mg L^-1^) | 54 | 14 | 102 | 115 |
| TS (mg L^-1^) | 1320 | 291 | 2080 | 687 |

**Table S2** Water quality parameters of the four kinds of water after reaction.

|  | Washing water from mixer trucks | The Qiantang River | Coal washing water | The Yellow River |
| --- | --- | --- | --- | --- |
| COD (mg L^-1^) | 21 | 20 | 17 | 17 |
| TDS (mg L^-1^) | 82 | 73 | 88 | 60 |
| TOC (mg L^-1^) | 1.2 | 0.8 | 1.2 | 0.6 |
| DOC (mg L^-1^) | 0.408 | 0.178 | 0.381 | 0.152 |
| HS (mg L^-1^) | <0.02 | <0.02 | <0.02 | <0.02 |
| BP (mg L^-1^) | 0.006 | <0.002 | <0.002 | <0.002 |
| pH | 7.3 | 7.4 | 7.6 | 7.2 |
| TP (mg L^-1^) | <0.01 | <0.01 | <0.01 | <0.01 |
| TH (mg L^-1^) | <5.005 | <5.005 | <5.005 | <5.005 |
| TN (mg L^-1^) | 0.54 | 0.44 | 1.73 | 0.27 |
| TSS (mg L^-1^) | 7 | 7 | 7 | 8 |
| TS (mg L^-1^) | 92 | 81 | 98 | 79 |

**References**

[1] a) G. Cheng, X. Liu, X. Song, X. Chen, W. Dai, R. Yuan, X. Fu, *Appl. Catal., B* **2020**, 277, 119196; b) C. Wang, W. Zhang, J. Wang, P. Xia, X. Duan, Q. He, I. Sirés, Z. Ye, *Appl. Catal., B* **2024**, 342, 123457.
